# *N*′-(4-Methyl­benzyl­idene)thio­phene-2-carbohydrazide

**DOI:** 10.1107/S1600536810017976

**Published:** 2010-05-22

**Authors:** Yu-Feng Li, Fang-Fang Jian

**Affiliations:** a Microscale Science Institute, Department of Chemistry and Chemical Engineering, Weifang University, Weifang 261061, People’s Republic of China; b Microscale Science Institute, Weifang University, Weifang 261061, People’s Republic of China

## Abstract

In the title compound, C_13_H_12_N_2_OS, the dihedral angle between the aromatic rings is 14.84 (17)°. In the crystal, inversion dimers linked by pairs of N—H⋯O hydrogen bonds generate *R*_2_^2^(8) loops.

## Related literature

For a related structure, see: Li & Jian (2010 ▶).
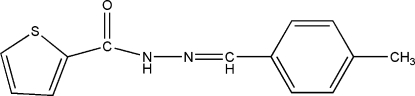


## Experimental

### 

#### Crystal data

C_13_H_12_N_2_OS
*M* *_r_* = 244.31
Monoclinic, 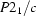

*a* = 14.920 (3) Å
*b* = 5.3976 (11) Å
*c* = 15.636 (3) Å
β = 105.87 (3)°
*V* = 1211.2 (4) Å^3^
*Z* = 4
Mo *K*α radiation
μ = 0.25 mm^−1^
*T* = 293 K
0.22 × 0.20 × 0.18 mm

#### Data collection

Bruker SMART CCD diffractometer
10416 measured reflections
2697 independent reflections
1759 reflections with *I* > 2σ(*I*)
*R*_int_ = 0.051

#### Refinement

*R*[*F* ^2^ > 2σ(*F* ^2^)] = 0.080
*wR*(*F* ^2^) = 0.275
*S* = 1.08
2697 reflections
154 parameters
H-atom parameters constrained
Δρ_max_ = 0.73 e Å^−3^
Δρ_min_ = −0.47 e Å^−3^

### 

Data collection: *SMART* (Bruker, 1997 ▶); cell refinement: *SAINT* (Bruker, 1997 ▶); data reduction: *SAINT*; program(s) used to solve structure: *SHELXS97* (Sheldrick, 2008 ▶); program(s) used to refine structure: *SHELXL97* (Sheldrick, 2008 ▶); molecular graphics: *SHELXTL* (Sheldrick, 2008 ▶); software used to prepare material for publication: *SHELXTL*.

## Supplementary Material

Crystal structure: contains datablocks global, I. DOI: 10.1107/S1600536810017976/hb5447sup1.cif

Structure factors: contains datablocks I. DOI: 10.1107/S1600536810017976/hb5447Isup2.hkl

Additional supplementary materials:  crystallographic information; 3D view; checkCIF report

## Figures and Tables

**Table 1:** Hydrogen-bond geometry (Å, °)

*D*—H⋯*A*	*D*—H	H⋯*A*	*D*⋯*A*	*D*—H⋯*A*
N2—H2*A*⋯O1^i^	0.86	2.07	2.919 (4)	170

Symmetry code: (i) 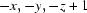.

**Fig. 1.**
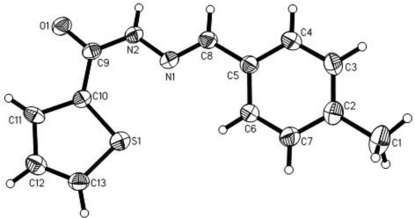
The structure of (I) showing 30% probability displacement ellipsoids.

## supplementary crystallographic information

### Experimental

A mixture of thiophene-2-carbohydrazide (0.10 mol) and 4-methylbenzaldehyde (0.10 mol) was stirred in refluxing ethanol (10 ml) for 4 h to afford the title compound (0.079 mol, yield 79%). Colourless blocks of (I) were obtained by recrystallization from ethanol at room temperature.

### Refinement

H atoms were fixed geometrically and allowed to ride on their attached atoms, with C—H distances = 0.93-0.97 Å; N—H = 0.86Å and with *U*_iso_(H) = 1.2*U*_eq_(C,*N*) or 1.5*U*_eq_(C_methyl_).

### Crystal data

**Table:**

C_13_H_12_N_2_OS	*F*(000) = 512
*M**_r_* = 244.31	*D*_x_ = 1.340 Mg m^−^^3^
Monoclinic, *P*2_1_/*c*	Mo *K*α radiation, λ = 0.71073 Å
Hall symbol: -P 2ybc	Cell parameters from 1759 reflections
*a* = 14.920 (3) Å	θ = 27.5–3.4°
*b* = 5.3976 (11) Å	µ = 0.25 mm^−^^1^
*c* = 15.636 (3) Å	*T* = 293 K
β = 105.87 (3)°	Block, colorless
*V* = 1211.2 (4) Å^3^	0.22 × 0.20 × 0.18 mm
*Z* = 4	

### Data collection

**Table:**

Bruker SMART CCD diffractometer	1759 reflections with *I* > 2σ(*I*)
Radiation source: fine-focus sealed tube	*R*_int_ = 0.051
graphite	θ_max_ = 27.5°, θ_min_ = 3.4°
phi and ω scans	*h* = −19→19
10416 measured reflections	*k* = −6→6
2697 independent reflections	*l* = −20→20

### Refinement

**Table:**

Refinement on *F*^2^	Primary atom site location: structure-invariant direct methods
Least-squares matrix: full	Secondary atom site location: difference Fourier map
*R*[*F*^2^ > 2σ(*F*^2^)] = 0.080	Hydrogen site location: inferred from neighbouring sites
*wR*(*F*^2^) = 0.275	H-atom parameters constrained
*S* = 1.08	*w* = 1/[σ^2^(*F*_o_^2^) + (0.1685*P*)^2^ + 0.1567*P*] where *P* = (*F*_o_^2^ + 2*F*_c_^2^)/3
2697 reflections	(Δ/σ)_max_ < 0.001
154 parameters	Δρ_max_ = 0.73 e Å^−^^3^
0 restraints	Δρ_min_ = −0.47 e Å^−^^3^

### Special details

**Table:**

Geometry. All esds (except the esd in the dihedral angle between two l.s. planes) are estimated using the full covariance matrix. The cell esds are taken into account individually in the estimation of esds in distances, angles and torsion angles; correlations between esds in cell parameters are only used when they are defined by crystal symmetry. An approximate (isotropic) treatment of cell esds is used for estimating esds involving l.s. planes.
Refinement. Refinement of *F*^2^ against ALL reflections. The weighted *R*-factor wR and goodness of fit *S* are based on *F*^2^, conventional *R*-factors *R* are based on *F*, with *F* set to zero for negative *F*^2^. The threshold expression of *F*^2^ > σ(*F*^2^) is used only for calculating *R*-factors(gt) etc. and is not relevant to the choice of reflections for refinement. *R*-factors based on *F*^2^ are statistically about twice as large as those based on *F*, and *R*- factors based on ALL data will be even larger.

### Fractional atomic coordinates and isotropic or equivalent isotropic displacement parameters (Å^2^)

**Table:**

	*x*	*y*	*z*	*U*_iso_*/*U*_eq_	
S1	0.20737 (7)	0.6743 (2)	0.48624 (6)	0.0649 (4)	
N1	0.17714 (18)	0.2992 (5)	0.59639 (17)	0.0481 (7)	
N2	0.09655 (17)	0.2160 (6)	0.53950 (16)	0.0507 (7)	
H2A	0.0658	0.1007	0.5569	0.061*	
O1	−0.00815 (15)	0.2169 (5)	0.40660 (15)	0.0576 (7)	
C9	0.0635 (2)	0.3089 (6)	0.4567 (2)	0.0461 (7)	
C8	0.2059 (2)	0.1867 (6)	0.6707 (2)	0.0506 (8)	
H8A	0.1713	0.0564	0.6841	0.061*	
C5	0.2924 (2)	0.2600 (6)	0.7348 (2)	0.0476 (7)	
C10	0.11121 (19)	0.5146 (6)	0.42646 (19)	0.0460 (7)	
C2	0.4649 (2)	0.3822 (8)	0.8565 (2)	0.0573 (9)	
C12	0.1361 (2)	0.8109 (7)	0.3262 (2)	0.0607 (10)	
H12A	0.1276	0.9021	0.2741	0.073*	
C7	0.4251 (2)	0.5308 (8)	0.7826 (2)	0.0636 (10)	
H7A	0.4562	0.6732	0.7732	0.076*	
C11	0.0757 (2)	0.6016 (6)	0.3332 (2)	0.0506 (8)	
H11A	0.0261	0.5375	0.2889	0.061*	
C3	0.4150 (3)	0.1770 (7)	0.8695 (2)	0.0665 (10)	
H3A	0.4388	0.0766	0.9188	0.080*	
C6	0.3420 (3)	0.4727 (8)	0.7240 (2)	0.0598 (9)	
H6A	0.3176	0.5765	0.6758	0.072*	
C4	0.3296 (3)	0.1174 (8)	0.8101 (2)	0.0631 (10)	
H4A	0.2969	−0.0203	0.8211	0.076*	
C13	0.2054 (2)	0.8631 (7)	0.4008 (2)	0.0582 (9)	
H13A	0.2477	0.9916	0.4041	0.070*	
C1	0.5603 (3)	0.4412 (11)	0.9168 (2)	0.0842 (14)	
H1B	0.5764	0.3217	0.9640	0.126*	
H1C	0.5598	0.6038	0.9415	0.126*	
H1D	0.6054	0.4355	0.8833	0.126*	

### Atomic displacement parameters (Å^2^)

**Table:**

	*U*^11^	*U*^22^	*U*^33^	*U*^12^	*U*^13^	*U*^23^
S1	0.0703 (7)	0.0624 (8)	0.0608 (6)	−0.0180 (4)	0.0157 (5)	−0.0037 (4)
N1	0.0482 (14)	0.0436 (16)	0.0523 (14)	−0.0058 (11)	0.0134 (11)	−0.0062 (12)
N2	0.0476 (14)	0.0518 (18)	0.0515 (15)	−0.0119 (12)	0.0119 (11)	−0.0025 (12)
O1	0.0464 (12)	0.0575 (16)	0.0636 (14)	−0.0100 (10)	0.0059 (10)	−0.0006 (12)
C9	0.0418 (15)	0.0428 (19)	0.0553 (18)	−0.0020 (12)	0.0160 (13)	−0.0049 (13)
C8	0.0563 (18)	0.049 (2)	0.0486 (17)	−0.0078 (14)	0.0182 (13)	−0.0002 (14)
C5	0.0579 (18)	0.0424 (19)	0.0443 (15)	−0.0017 (14)	0.0170 (13)	−0.0032 (13)
C10	0.0402 (15)	0.0450 (19)	0.0521 (16)	0.0006 (12)	0.0114 (12)	−0.0028 (13)
C2	0.0542 (18)	0.071 (3)	0.0485 (17)	0.0056 (16)	0.0169 (14)	−0.0091 (16)
C12	0.0487 (18)	0.067 (3)	0.067 (2)	0.0068 (15)	0.0157 (15)	0.0172 (18)
C7	0.063 (2)	0.070 (3)	0.0566 (19)	−0.0181 (18)	0.0147 (15)	−0.0001 (18)
C11	0.0444 (16)	0.048 (2)	0.0649 (19)	0.0041 (13)	0.0249 (14)	0.0159 (15)
C3	0.077 (2)	0.063 (3)	0.052 (2)	0.0041 (19)	0.0038 (16)	0.0088 (17)
C6	0.069 (2)	0.055 (2)	0.0504 (17)	−0.0126 (17)	0.0085 (15)	0.0067 (16)
C4	0.078 (2)	0.053 (2)	0.057 (2)	−0.0079 (18)	0.0159 (17)	0.0089 (16)
C13	0.062 (2)	0.046 (2)	0.065 (2)	−0.0056 (15)	0.0150 (16)	0.0037 (16)
C1	0.058 (2)	0.131 (5)	0.059 (2)	0.001 (2)	0.0076 (17)	−0.013 (3)

### Geometric parameters (Å, °)

**Table:**

S1—C13	1.673 (3)	C2—C1	1.511 (5)
S1—C10	1.715 (3)	C12—C13	1.360 (5)
N1—C8	1.277 (4)	C12—C11	1.468 (5)
N1—N2	1.362 (3)	C12—H12A	0.9300
N2—C9	1.350 (4)	C7—C6	1.362 (5)
N2—H2A	0.8600	C7—H7A	0.9300
O1—C9	1.242 (4)	C11—H11A	0.9300
C9—C10	1.465 (4)	C3—C4	1.394 (5)
C8—C5	1.455 (4)	C3—H3A	0.9300
C8—H8A	0.9300	C6—H6A	0.9300
C5—C4	1.389 (5)	C4—H4A	0.9300
C5—C6	1.400 (5)	C13—H13A	0.9300
C10—C11	1.486 (4)	C1—H1B	0.9600
C2—C3	1.380 (5)	C1—H1C	0.9600
C2—C7	1.399 (5)	C1—H1D	0.9600
			
C13—S1—C10	92.39 (16)	C6—C7—H7A	119.1
C8—N1—N2	117.1 (3)	C2—C7—H7A	119.1
C9—N2—N1	122.0 (3)	C12—C11—C10	104.7 (3)
C9—N2—H2A	119.0	C12—C11—H11A	127.6
N1—N2—H2A	119.0	C10—C11—H11A	127.6
O1—C9—N2	118.8 (3)	C2—C3—C4	121.3 (3)
O1—C9—C10	120.7 (3)	C2—C3—H3A	119.4
N2—C9—C10	120.5 (3)	C4—C3—H3A	119.4
N1—C8—C5	120.8 (3)	C7—C6—C5	121.5 (3)
N1—C8—H8A	119.6	C7—C6—H6A	119.3
C5—C8—H8A	119.6	C5—C6—H6A	119.3
C4—C5—C6	117.0 (3)	C5—C4—C3	121.2 (4)
C4—C5—C8	120.4 (3)	C5—C4—H4A	119.4
C6—C5—C8	122.6 (3)	C3—C4—H4A	119.4
C9—C10—C11	118.8 (3)	C12—C13—S1	113.7 (3)
C9—C10—S1	127.8 (2)	C12—C13—H13A	123.1
C11—C10—S1	113.4 (2)	S1—C13—H13A	123.1
C3—C2—C7	117.2 (3)	C2—C1—H1B	109.5
C3—C2—C1	122.1 (4)	C2—C1—H1C	109.5
C7—C2—C1	120.6 (4)	H1B—C1—H1C	109.5
C13—C12—C11	115.7 (3)	C2—C1—H1D	109.5
C13—C12—H12A	122.1	H1B—C1—H1D	109.5
C11—C12—H12A	122.1	H1C—C1—H1D	109.5
C6—C7—C2	121.8 (4)		

### Hydrogen-bond geometry (Å, °)

**Table:**

*D*—H···*A*	*D*—H	H···*A*	*D*···*A*	*D*—H···*A*
N2—H2A···O1^i^	0.86	2.07	2.919 (4)	170

Symmetry codes: (i) −*x*, −*y*, −*z*+1.
